# Open or closed abdomen post laparotomy to control severe abdominal sepsis: a survival analysis

**DOI:** 10.1590/0100-6991e-20243595-en

**Published:** 2024-04-17

**Authors:** IMAD SHEHADEH, LUCIANO DE ANDRADE, ARIANA IEDA LIMA FERREIRA DA SILVA, PEDRO HENRIQUE IORA, EDUARDO FALCO KNAUT, GIORDANNA CHIQUETO DUARTE, CARLOS EDMUNDO RODRIGUES FONTES

**Affiliations:** 1 - Hospital Universitário Regional de Maringá, Departamento de Cirurgia Geral - Maringá - PR - Brasil; 2 - Universidade Estadual de Maringá, Departamento de Medicina - Maringá - PR - Brasil; 3 - Universidade Estadual de Maringá, Programa de Mestrado em Gestão Tecnologia e Inovação em Urgência e Emergência - Maringá - PR - Brasil

**Keywords:** Sepsis, Open Abdomen Treatment, Negative-Pressure Wound Therapy, Sepse, Tratamento de Abdome Aberto, Tratamento de Ferimentos com Pressão Negativa

## Abstract

**Introduction:**

severe abdominal sepsis, accompained by diffuse peritonitis, poses a significant challenge for most surgeons. It often requires repetitive surgical interventions, leading to complications and resulting in high morbidity and mortality rates. The open abdomen technique, facilitated by applying a negative-pressure wound therapy (NPWT), reduces the duration of the initial surgical procedure, minimizes the accumulation of secretions and inflammatory mediators in the abdominal cavity and lowers the risk of abdominal compartment syndrome and its associated complications. Another approach is primary closure of the abdominal aponeurosis, which involves suturing the layers of the abdominal wall.

**Methods:**

the objective of this study is to conduct a survival analysis comparing the treatment of severe abdominal sepsis using open abdomen technique versus primary closure after laparotomy in a public hospital in the South of Brazil. We utilized data extracted from electronic medical records to perform both descriptive and survival analysis, employing the Kaplan-Meier curve and a log-rank test.

**Results:**

the study sample encompassed 75 laparotomies conducted over a span of 5 years, with 40 cases employing NPWT and 35 cases utilizing primary closure. The overall mortality rate observed was 55%. Notably, survival rates did not exhibit statistical significance when comparing the two methods, even after stratifying the data into separate analysis groups for each technique.

**Conclusion:**

recent publications on this subject have reported some favorable outcomes associated with the open abdomen technique underscoring the pressing need for a standardized approach to managing patients with severe, complicated abdominal sepsis.

## RESUMO

**Introdução:**

a sepse abdominal grave, com peritonite difusa, é um grande desafio para o cirurgião geral, sendo muito frequente as reintervenções cirúrgicas e complicações desta doença, que apresenta morbimortalidade elevada. A proposta do abdome aberto, aplicando-se a terapia por pressão negativa (TPN), reduz o tempo operatório da primeira abordagem cirúrgica, reduz o acúmulo de secreções e mediadores inflamatórios no sítio abdominal, diminui a possibilidade de síndrome compartimental abdominal e suas complicações. A outra técnica é a síntese primária, quando é optado por fechar a parede por completo.

**Métodos:**

o objetivo do estudo foi realizar uma análise de sobrevivência comparando os tratamentos de sepse intra-abdominal severa com Abdome Aberto e Fechado após laparotomia em um hospital universitário no sul do Brasil. As variáveis foram obtidas a partir de prontuários eletrônicos de pacientes submetidos à intervenção cirúrgica e realizou-se uma análise descritiva e de sobrevivência usando a curva de Kaplan-Meier e o teste de log-rank.

**Resultados:**

a amostra incluiu 75 laparotomias em 5 anos (40 TPN e 35 sínteses primárias), com mortalidade de 55%. A sobrevida entre os métodos de fechamento não demonstrou significância estatística, mesmo após a divisão em um grupo de análise de cada técnica.

**Conclusão:**

recentes publicações destacam resultados favoráveis do abdome aberto, enfatizando a necessidade urgente de uma sistematização na assistência de pacientes com sepse abdominal complicada grave.

## INTRODUCTION

Sepsis is one of the main causes of death in the world^1^ ^,^ ^2^, and intra-abdominal sepsis is the second most common form of sepsis^3^. Generalized peritonitis, which then progress to severe complicated abdominal sepsis (SCAS), represents one of the most challenging clinical situations that surgeons face in their daily care routine. It can quickly progress to shock and failure of multiple organs and systems, leading to relevant morbidity and mortality rates^4^ ^-^ ^7^.

This condition very commonly requires reoperations to review the abdominal cavity to eliminate persistent or recurrent peritonitis or to treat complications from the progression of infection, even for those patients who receive expanded antimicrobial therapy and adequate clinical support after an initial surgical approach^8^ ^-^ ^10^.

One of the therapeutic strategies is treatment with an open abdomen, without primary closure of the abdominal wall at the end of a laparotomy, applying negative pressure therapy (NPT) to remove the accumulation of fecal, enteric, purulent, inflammatory, and/or infectious ascites, to try and control the septic focus^4^ ^,^ ^6^ ^,^ ^9^ ^,^ ^11^ ^-^ ^13^. The other strategy is the primary synthesis of the cavity at the end of the first operative approach, which is part of the routine of any abdominal surgery.

Publications on the subject demonstrate results favorable to NPT^14^ ^-^ ^17^. Complications such as enteric fistula and inability to close the aponeurosis, with progression to incisional hernia, were more frequent when the primary synthesis of the abdominal wall was not performed and the abdomen was left open in a peritoneostomy, without negative pressure applied^6,8,18 -21^.

To reduce complications and harm to these patients, diagnosis and appropriate therapy must be carried out as soon as possible^22^. The delay in decision-making and/or in referring the patient to a specialized center causes morbidity and mortality to increase significantly^22^. Therefore, reference hospitals must be prepared for a surgical approach, in addition to providing excellent clinical and hemodynamic support for patients with SCAS.

A global consensus on the best approach has not yet been defined, that is, primary synthesis of the aponeurosis in the first operation or performance of NPT. Although primary synthesis is still used for treatment, many studies have already demonstrated satisfactory results with NPT^4^ ^,^ ^5^ ^,^ ^14^ ^-^ ^16^ ^,^ ^23^ ^,^ ^24^ ^-^ ^27^. Current research shows that this therapeutic approach, especially after the development and improvement of advanced therapies, has been a reliable and feasible option, providing greater safety to the abdominal viscera and greater control of the spread of inflammatory mediators of abdominal sepsis^14^ ^,^ ^23^.

The objective of this study was to carry out a survival analysis of patients treated at a university hospital in southern Brazil, comparing treatments for severe intra-abdominal sepsis with Barker-type, open-abdomen negative pressure therapy (NPT) or with primary aponeurosis synthesis after laparotomy. 

## METHODS

### Study design and location

We conducted an observational, retrospective research, based on secondary data obtained from electronic medical records of patients with diffuse peritonitis that progressed to SCAS treated at the Hospital Universitário Regional de Maringá (HURM) between 2017 and 2021.

HURM is a reference center for more than 115 counties in the northwestern macro-region of the state of Paraná, Southern Brazil, serving a population of approximately two million for various causes. Among these, acute abdomen stands out, most patients being referred to the tertiary hospital from health services that lack the capacity for definitive treatment.

### Inclusion and exclusion criteria

We included individuals over eighteen years old with known or suspected infectious cause of abdominal focus and one or more signs of hemodynamic instability (volume-refractory hypotension, tachypnea, tachycardia, lability of body temperature, change in level of consciousness, oliguria, cold extremities, and/or thin pulses with signs of poor perfusion, among others)^1^ ^-^ ^3^ ^,^ ^5^ ^,^ ^7^, with intraoperative evidence of purulent spillage and presence of free enteric content in the abdominal cavity, who underwent closure with primary aponeurosis synthesis or NPT^27^.

The NPT used in the service was the Barker technique, which consists of a fenestrated, non-adherent polyethylene sheet placed over the viscera and covered with sterile surgical pads. Two surgical drains are positioned between the pads, the abdomen is sealed with a large adhesive dressing, and the drains are connected to a continuous suction system^28^.

Exclusion criteria were pregnancy, trauma, laparoscopy, inability to close the cavity due to undue tension or inducing abdominal hypertension, and uncontrollable bleeding. We also excluded patients with data considered insufficient for analysis or barely present in the medical records, minimizing potential bias.

Due to high-cost issues, no commercial dressings were used in the hospital service for the treatment of severe intra-abdominal sepsis.

### Data source

Secondary data collected from patients’ electronic medical records were stored in an Microsoft Excel spreadsheet. Subsequently, they were analyzed in a descriptive and inferential way using the R software. The variables used in the study include demographics (age and sex), type of procedure and duration, ASA score (American Society of Anesthesiologist), surgical indication, time of onset of symptoms, closure technique of the first approach, duration of NPT in days, number of surgical interventions, number of complications, days of intensive care, time from arrival to outcome, and interval in days between the procedures.

### Data analysis

After the descriptive analysis, we used the chi-square statistical test to make comparisons between the two abdominal wall closure techniques as to the described variables of interest, adopting a significance level of 5% (p≤0.05).

Survival analysis was performed using the Kaplan-Meier method. Initially, an overall survival curve was constructed, representing patient survival throughout hospitalization. Then, a second curve was created to compare patients who underwent the primary aponeurosis closure technique with those who received NPT, applying the log-rank test. Furthermore, for a more comprehensive analysis, we established a categorization criterion that involved the definition of a specific therapeutic group for each of the two surgical techniques studied. This distinction was made as follows: if in the first surgery in which primary synthesis was adopted as the closure technique patients subsequently required a second surgical intervention, they were considered as members of the group. This occurred when the initial approach was not sufficient to resolve the problem. On the other hand, if in the first surgery in which NPT was used and in the subsequent intervention it was not possible to close the abdominal wall, these cases were also included in the analysis of this portion of the sample. This indicates that the initial technique did not achieve the desired result.

The final manuscript of the present study followed the STROBE (Strengthening the Reporting of Observational Studies in Epidemiology) guidelines, which guarantee transparent reporting and are considered the standard for observational studies^30^.

The project was approved by the Academic Activities Regulation Commission (COREA) of HURM (n° 059/2020) and by the Permanent Ethics Committee on Research with human beings of the State University of Maringá (COPEP/UEM) - (CAAE: 63638822.4.0000.0104).

## RESULTS

There were 75 laparotomies for abdominal infectious causes during the five-year period, 48 male patients (64%) and 27 females (36%). The average age was 59.52 years, with a standard deviation of 17.32 and a median of 61.

The indications for surgical treatment were divided into the different types of acute abdomen, such as perforating, inflammatory, obstructive, and ischemic (Table 1). There was no hemorrhagic abdomen within the sample.

**Table 1:** Distribution of patients according to the type of acute abdomen.

Acute abdomen type	Number of patients	Percentage
Perforating	33	44%
Inflammatory	26	34.7%
Obstructive	9	12%
Ischemic	7	9.3%

The duration of the procedures, in minutes, had an average of approximately 180, with a minimum of 60 and a maximum of 480, and a median of 175 minutes. Most patients were ASA 2 (17), 3 (30), and 4 (22), the remainder being six cases. The time from onset of symptoms to the operation in question resulted in an average of three days, indicating a rapid evolution of symptoms.

In the medical records analyzed, we found two main techniques for closing the abdominal wall for the treatment of SCAS. In total, there were 40 NPTs implemented using the Barker technique and 35 primary syntheses. Of the patients with a known outcome, excluding those who were transferred or evaded medical care, 31 (45%) were discharged and 38 (55%) died.

Patients who required re-approach were typically for review of the cavity and lavage, in addition to evaluation of complications. The distribution approximated an average of three operations from the first intervention and a standard deviation of 2.76. The main surgical complications were fistulas (29.3%), surgical wound infection (9.3%), bowel loop ischemia (8%), intra-abdominal abscess (8%), intra-abdominal hematoma (5.3%), evisceration (5.3%), and abdominal wall bleeding (4%). Furthermore, other less frequent surgical complications were documented, such as incisional hernia, colostomy necrosis, seroma, adhesions, and extremity ischemia (1.3%).

Among fistula-type complications, enteric fistula was the most frequent, represented by 47.8% of cases, followed by colonic one, in 21.7% of cases (Table 2).

**Table 2:** Description of the types of fistulas.

Types of fistula	Number of patients	Percentage
Not classified	4	17.4%
Colonic	5	21.7%
Enteric	11	47.8%
Duodenal	2	8.7%
Pancreatic	1	4.4%

In patients in whom NPT was used, its average duration was 6.66 days, standard deviation of 8.55, and median of 3, with a maximum of 39 days. The average length of stay in an intensive care environment was 14.73 days, with a standard deviation of 18.86. The time from NPT to outcome was quite variable, with a mean of 20.19 days, a median of 18, and a standard deviation of 16.94 days.

Table 3 demonstrates the distribution of variables by type of treatment instituted. The chi-square test, based on the distribution between groups and associated variables, such as sex, ASA, and number of complications, did not show statistical significance. However, age presented a significant p-value, demonstrating homogeneous distribution between age groups for patients undergoing NPT versus primary synthesis.

**Table 3:** Comparison of variables by therapy group.

Variable	NPT	Primary Synthesis	Chi-Square
Sex			
Male	26	21	0.698
Female	14	14	
Age group			
20-40	10	1	0.024
41-60	10	14	
61-80	16	13	
81-100	4	7	
ASA			
1	1	2	0.493
2	9	9	
3	14	15	
4	12	9	
5	4	0	
Number of complications			
0	22	17	0.512
1	9	7	
2	5	9	
3	4	2	

**Figure 1:**
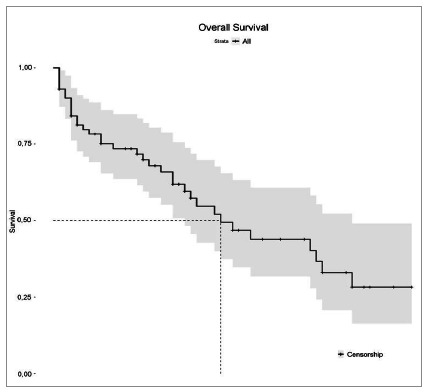
Univariate analysis of survival after the first intervention.

**Figure 2:**
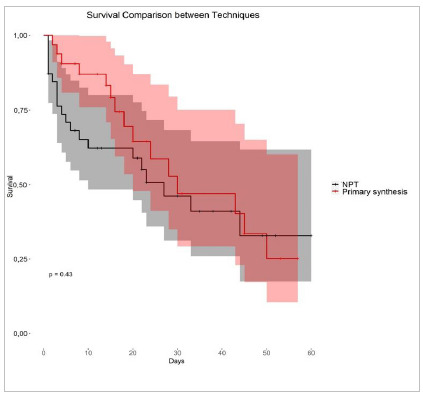
Survival time until outcome according to the technique used in the first intervention.

Patient survival was analyzed with the Kaplan-Meier method, allowing a clear observation of the relationship between a lower chance of survival and increased length of stay (Figures 1 and 2). This downward trend, although relatively constant, did not allow the identification of a clear cut-off period.

Figure 1 demonstrates the overall survival of patients throughout hospitalization. The median survival occurs approximately on the 28^th^ day, so that at the end of the first month of hospitalization, half of the patients remained alive, not considering the patients who were discharged, represented by the crosses (+) in the graph.

When comparing open abdomen with primary synthesis (Figure 2), the log-rank test resulted in a p-value of 0.43, not statistically significant. Therefore, there is insufficient evidence to reject the null hypothesis that the two techniques have the same effect on survival. Furthermore, we can observe a similarity in the curves, with a slight advantage for primary closure in the initial days after surgery. For example, NPT reached 50% mortality just after the 20^th^ day, while primary synthesis did it close to the 30^th^ day.

**Figure 3:**
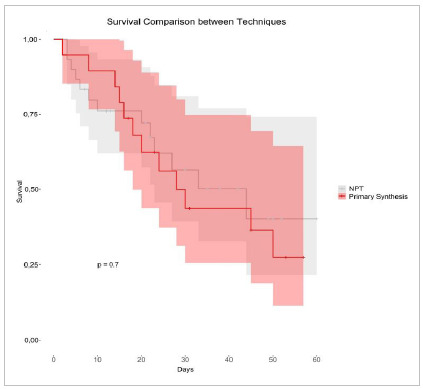
Survival curve according to the closure technique used.

Despite the lack of statistical significance when comparing the treatments (Figure 2), when exploring survival between the techniques in more depth (Figure 3), we observed that the survival analysis curves for the two different approaches are remarkably similar, with a p-value of 0.7, confirming the lack of statistically significant difference between them. Although at times patients with prolonged hospitalization on NPT seem to have a favorable evolution, mainly from the second week of hospitalization on, it is important to highlight that the curves cross on three distinct occasions during the period studied. This points to a continuous oscillation between the approaches and any conclusive statement about the superiority of one over the other would be premature based on these results, so a data set with a larger number of participants would allow for a more robust and conclusive analysis.

## DISCUSSION

During the process of searching and analyzing the cases brought to light in this study, we observed that the general surgery service at the hospital where the study was conducted already applies Barker-type NPT to patients diagnosed with SCAS. However, we did not identify a set of pre-defined, standardized, systematized actions. Intraoperative decisions were chosen based on the personal experience of the surgeon responsible for each surgical procedure, in addition to monitoring and clinical conduct during hospital stay.

As for stratification by cause, we found a high incidence of perforating acute abdomen. Perhaps due to the delay in transferring a patient to the reference center or even due to the epidemiological profile of the sample, with patients with various comorbidities and advanced age, gastroduodenal ulcers, for example, prevailed. Another important point is the absence of acute hemorrhagic abdomen, which is easily explained, as trauma was considered an exclusion criterion.

Among the complications detected, the sample deviates from some numbers frequently reported in other services. The Atema^20^ meta-analysis brought variations from 5.7% to 17.2% for fistulas. These values, although focused on the abdominal wall closure technique, are lower than the 29.3% in our institution. Other complications are less common and are consistent with other surveys^20^ ^,^ ^21^.

The approach to sepsis requires effective decision-making as soon as patient care begins^31^. The treatment of abdominal sepsis has the surgical approach as the main therapeutic pillar. It also includes immediate elimination of the infectious focus, with intensive resuscitation support and antimicrobial therapy, in addition to reoperations^15^ ^,^ ^19^.

In the literature, there is still no consensus on which patients should undergo relaparotomy^8^ ^,^ ^15^. The decision is often challenging and difficult, especially when faced with critically ill patients with non-specific signs and symptoms of sepsis. The need and timing of relaparotomy is also still very subjective^10^, as without standardization of conduct, optimized results may not be achieved.

In a large Dutch study conducted by Van Ruler^8^, 42% of patients required relaparotomy to control persistent or suspected peritonitis. Unusually, 31% of these patients had a negative laparotomy. They observed that in the group of critically ill patients, mortality was not lower with planned relaparotomy, contrary to what is currently widely accepted. The results, then, concluded that on-demand laparotomy versus planned laparotomy really was the most rational approach at the time. However, a criticism to consider in understanding surgical source control is that Van Ruler8 did not use a contemporary open abdomen approach in either arm of his study and that the abdominal aponeurosis was formally closed in both groups.

Aspects other than the mortality rate are under discussion. Some studies indicate that on-demand laparotomy substantially reduces the number of relaparotomies, the need for Intensive Care Unit (ICU), and medical costs^32^. According to Scriba^32^, the ICU admission rate was 45% lower in on-demand laparotomy.

A meta-analysis of retrospective studies by Lamme^33^ and a subsequent randomized clinical trial by Van Ruler^8^ (aptly called the “RELAP” trial) both concluded that planned relaparotomy does not bring a survival advantage, may in fact increase morbidity, and leads to significant increases in healthcare costs.

Despite the advancement and improvement of intensive therapy^31^, isolated pharmacological therapies are not the answer to controlling generalized infection and organ dysfunction. Several trials in this regard were proposed to contain post-infectious inflammation, proving, however, to be extremely expensive and frustrating, without beneficial results for patients^34^ ^,^ ^35^. 

The results from the study’s institution, from 2017 to 2021, did not show statistically significant outcomes when comparing the primary closure of the aponeurosis and the application of a vacuum dressing for the treatment of SCAS. We also observed, for patients undergoing NPT in the first operation, a lack of standardization regarding the best time for re-approaches, the external appearance of the dressing often being the reason for the decision on when to perform a new procedure. Overall, the survival curves demonstrated limited results, but with some important points of discussion. The individual general clinical picture appears to be a primary and independent factor in mortality. However, if the patient survives long enough for long-term assessment, little difference is noted in the two techniques used. Therefore, it is important to highlight that, in agreement with their condition, critically ill patient groups undergo more interventions, the need for early re-approach being a clear factor in indicating NPT.

This proves that establishing a systematization for the treatment of patients with SCAS is essential to obtain optimized results that follow updated research on the topic^15^ ^,^ ^17^ ^,^ ^36^ ^-^ ^38^.

The open abdomen (OA) strategy in general surgery has been increasingly reported in uncontrolled series as a potentially beneficial option for patients with severe complicated abdominal sepsis^5^ ^,^ ^8^ ^,^ ^9^ ^,^ ^12^ ^,^ ^13^ ^,^ ^39^. This therapeutic approach can allow identification of new accumulation of secretions, as well drainage of any residual infection, control of any persistent source of infection, effective removal of peritoneal fluid rich in inflammatory biomediators, prophylaxis against the development of abdominal compartment syndrome, and safe evaluation of previous gastrointestinal anastomoses^5^.

Although more randomized controlled trials are needed, meta-analyses conducted by a group of researchers from Canada^17^ and Amsterdam^15^ concluded that treatment with negative pressure wound therapy appears to be the safest and most effective abdominal management technique currently available.

Kirkpatrick conducted a prospective, randomized, controlled trial addressing this question, the Intraperitoneal Vacuum Trial^14^, in Calgary, Alberta. After careful patient selection and follow-up, the 90-day survival rate improved in the group undergoing active negative pressure therapy (hazard ratio, 0.32, 95% CI 0.11-0.93, p=0.04)^14^.

Evidently, care for these seriously ill patients is multidisciplinary^31^. Intensive clinical and surgical care, comprehensive antibiotic therapy, nutrition, physiotherapy, psychological support, and assistance to families in short form the foundation for a favorable evolution of patients affected by SCAS.

The present study has limitations regarding the selection of the therapy proposed for each patient, which was mostly at the discretion of the surgeon during the first approach. The authors propose, in the future, the implementation of a protocol with objective criteria for choosing between NPT or primary synthesis of the aponeurosis on a case-by-case basis. The development of such a protocol requires previous incidence studies and situational analysis in relation to NPT in the hospital, this work being the first developed in the service.

The use of retrospective data generated gaps regarding the severity of each patient: collection via electronic medical records is subject to heterogeneity in data filling, exam requests, medication prescription, and intensive care for each person on duty upon patient admission and during follow-up in an intensive environment. The authors recognize this limitation and suggest, for future studies, the standardization of data input in medical records through a specific questionnaire for SCAS to be filled out during admission and hospitalization, with the filling of severity and prognosis scales and adequate stratification of the severity of each patient.

The future perspective of this work will be to implement a protocol for approaching and monitoring patients diagnosed with SCAS in the study’s institution. To this end, knowledge of the current state of management and pre-protocol prognosis of affected patients is fundamental, serving as a foundation for the development of future research.

## CONCLUSION

When applying a survival analysis of patients with SCAS divided into the therapeutic groups of primary closure of the aponeurosis and application of NPT treated at the institution in question, we found no statistically significant difference in relation to the best therapeutic approach. High mortality prevailed at the end of the first surgery for both groups.
